# Type 3 Secretion System (T3SS) of *Bradyrhizobium* sp. DOA9 and Its Roles in Legume Symbiosis and Rice Endophytic Association

**DOI:** 10.3389/fmicb.2017.01810

**Published:** 2017-09-20

**Authors:** Pongpan Songwattana, Rujirek Noisangiam, Kamonluck Teamtisong, Janpen Prakamhang, Albin Teulet, Panlada Tittabutr, Pongdet Piromyou, Nantakorn Boonkerd, Eric Giraud, Neung Teaumroong

**Affiliations:** ^1^School of Biotechnology, Institute of Agricultural Technology, Suranaree University of Technology Nakhon Ratchasima, Thailand; ^2^National Bureau of Agricultural Commodity and Food Standards, Ministry of Agriculture and Cooperatives Bangkok, Thailand; ^3^The Center for Scientific and Technological Equipment, Suranaree University of Technology Nakhon Ratchasima, Thailand; ^4^Department of Applied Biology, Faculty of Sciences and Liberal Arts, Rajamangala University of Technology Isan Nakhon Ratchasima, Thailand; ^5^Institut de Recherche pour le Développement, LSTM, UMR IRD/SupAgro/INRA/Univ. Montpellier/CIRAD Montpellier, France

**Keywords:** type 3 secretion system, effector proteins, *Bradyrhizobium*, legume, symbiosis

## Abstract

The *Bradyrhizobium* sp. DOA9 strain isolated from a paddy field has the ability to nodulate a wide spectrum of legumes. Unlike other bradyrhizobia, this strain has a symbiotic plasmid harboring *nod*, *nif*, and type 3 secretion system (T3SS) genes. This T3SS cluster contains all the genes necessary for the formation of the secretory apparatus and the transcriptional activator (TtsI), which is preceded by a *nod*-box motif. An *in silico* search predicted 14 effectors putatively translocated by this T3SS machinery. In this study, we explored the role of the T3SS in the symbiotic performance of DOA9 by evaluating the ability of a T3SS mutant (Ω*rhcN*) to nodulate legumes belonging to Dalbergioid, Millettioid, and Genistoid tribes. Among the nine species tested, four (*Arachis hypogea*, *Vigna radiata*, *Crotalaria juncea*, and *Macroptilium atropurpureum*) responded positively to the *rhcN* mutation (ranging from suppression of plant defense reactions, an increase in the number of nodules and a dramatic improvement in nodule development and infection), one (*Stylosanthes hamata*) responded negatively (fewer nodules and less nitrogen fixation) and four species (*Aeschynomene americana*, *Aeschynomene afraspera*, *Indigofera tinctoria*, and *Desmodium tortuosum*) displayed no phenotype. We also tested the role of the T3SS in the ability of the DOA9 strain to endophytically colonize rice roots, but detected no effect of the T3SS mutation, in contrast to what was previously reported in the *Bradyrhizobium* SUTN9-2 strain. Taken together, these data indicate that DOA9 contains a functional T3SS that interferes with the ability of the strain to interact symbiotically with legumes but not with rice.

## Introduction

The establishment of the legume–rhizobium symbiosis relies on the exchange of diffusible signal molecules between the two partners. The first important bacterial signals are lipochitooligosaccharides (LCOs) called Nod factors (NFs) that are synthetized and secreted after activation of bacterial nodulation (*nod*) genes by the transcriptional regulator protein NodD in specific response to host flavonoids. In return, perception of the NFs signal by the plant triggers nodule organogenesis and bacterial infection processes (Oldroyd et al., 2011). Besides NFs, other bacterial components are important for the successful establishment of the symbiosis, including exopolysaccharides (EPS), lipopolysaccharides (LPS), capsular polysaccharides (KPS), and cyclic β-glucans (Gibson et al., 2008; Janczarek et al., 2015). These bacterial compounds can also act as a symbiotic signal or permit to avoid plant immune responses (Gourion et al., 2015; Kawaharada et al., 2015).

In addition, some rhizobia use a type 3 secretion system (T3SS), which shares homology with the T3SS apparatus of plant and animal pathogenic bacteria, to translocate some putative effectors into the host cell, named nodulation outer proteins (Nops). Those putative effector proteins may have contrasting effects on nodulation depending on the host plant (Staehelin and Krishnan, 2015). Some secreted effector proteins can play a positive role in the establishment and maintenance of symbiosis by suppressing plant defense reactions thereby facilitating the bacterial infection (Bartsev et al., 2004; Jiménez-Guerrero et al., 2015). Conversely, secreted Nop proteins can be recognized by plant receptor proteins [resistant (R) proteins] leading to effector-triggered immunity, which blocks infection and nodulation (Yang et al., 2010; Yasuda et al., 2016).

It is important to indicate that the term Nop has been initially introduced by Marie et al. (2001) to mirror the nomenclature of *Yersinia* outer proteins (Yops) and that the first identified Nops was done by characterizing the proteins secreted in *Sinorhizobium* NGR234 culture supernatants in a NodD1- and a flavonoid-dependent manner (Marie et al., 2003). As consequence, this term has been used to name some components of the extracellular appendages or pili of the T3SS, i.e., NopA, NopB, and NopX but also effector proteins secreted through the T3SS that are translocated to the interior of the host cell, i.e., NopL, NopE, and NopC. In this paper, we will consider the term Nop to speak about the effector proteins translocated into the host cell.

The involvment of the T3SS in the establishement of the symbiosis is supported by two facts: (i) the genes that code for T3SS machinery and some Nops are found in the same region as the *nod* and *nif* genes (on a symbiotic island or a symbiotic plasmid) and (ii) expression of the T3SS genes is also controlled via a regulatory cascade involving NodD. In the latter case, NodD regulates, in a flavonoid-dependent manner, the expression of the transcriptional activator (TtsI), which, in return, activates the expression of T3SS genes by binding to their promoter region at the level of a specific motif, called *tts*-box (Krause et al., 2002; Tampakaki, 2014).

The importance of T3SS in the modulation of the host spectrum has been extensively demonstrated in several rhizobial strains (*Sinorhizobium* sp. NGR234, *S. fredii* USDA257, *S. fredii* HH103, *Mesorhizobium loti* MAFF303099, *Bradyrhizobium diazoefficiens* USDA110, *B. elkanii* USDA61) (Viprey et al., 1998; Krause et al., 2002; Krishnan et al., 2003; de Lyra Mdo et al., 2006; López-Baena et al., 2009; Okazaki et al., 2009, 2010). More recently, some non-photosynthetic *Bradyrhizobium* strains have been shown to be able to nodulate some legume plants (*Glycine max* cv. Enrei and several *Aeschynomene* species) in the absence of NF synthesis but in these cases, nodulation requires a functional T3SS. This points to the existence of an alternative Nod-independent symbiotic pathway in which some Nop effectors can directly activate the nodulation signaling pathway (Okazaki et al., 2013, 2016). Moreover, in *Bradyrhizobium* sp. SUTN9-2, T3SS has been reported to be an important determinant of rice-endophyte colonization (Piromyou et al., 2015b). Taken together, these data show that the role played by the T3SS in rhizobia during the interaction with their host plants is more important and broader than previously believed.

The non-photosynthetic *Bradyrhizobium* sp. strain DOA9 was originally isolated from a paddy field in Thailand using *Aeschynomene americana* as trap plant (Noisangiam et al., 2012). In addition to its ability to endophytically colonize rice roots, this strain has been shown to be able to induce the formation of nodules in a large spectrum of legume hosts (Teamtisong et al., 2014). Unlike other bradyrhizobia, the DOA9 is the only strain yet identified to possess a symbiotic plasmid that harbors nitrogen fixation (*nif*), nodulation (*nod*), and T3SS genes (Okazaki et al., 2015). In this study, we examined the role of T3SS in the DOA9 strain by testing the symbiotic performance of a T3SS mutant (Ω*rhcN*) on various legume species as well as on rice.

## Materials and Methods

### Bacterial Strains and Culture Conditions

The bacterial strains used in this study are listed in **Table 1**. *Bradyrhizobium* sp. strain DOA9 and its Ω*rhcN* derivative were grown in YM medium (Vincent, 1970) at 28°C. *Escherichia coli* strains were cultured at 37°C in LB medium (Sambrook et al., 2001). The media were supplemented with antibiotics at the following concentrations when appropriate: 200 μg/mL streptomycin (Sm), 20 μg/mL nalidixic acid (Nal), and 50 μg/mL kanamycin (Km).

**Table 1:** Bacterial strains, plasmids used, and plants tested in this study.

	Relevant characteristics	Source or reference
**Strains**		
*Bradyrhizobium* sp. DOA9	Isolated from paddy field using *A. americana* as trap legume	Noisangiam et al., 2012
Ω*rhcN*	*rhcN* mutant of DOA9 strain obtained by integration of pVO155-Sm-npt2-gfp; Sm^r^ Sp^r^ Km^r^	This study
*Escherichia coli* S17-1	hsdR pro thi (RP4-2 km::Tn7 tc::Mu)	
**Plasmid**		
pVO155-Sm-npt2-gfp	pUC119-derived suicide vector with *gusA* gene, GFP, and Sm^r^ cassette, Km^R^ Sm^r^ Sp^r^	Wongdee et al., 2016
pMG103-npt2-Sm-npt2-gfp	Cloning vector harboring *gfp* gene under the control of the constitutive *npt2* promoter; Sm^r^ Sp^r^ Km^r^	Bonaldi et al., 2010


### Construction of the Reporter and Mutant Strains

The single cross-homologous recombination technique was used to construct the insertion mutant in the structural type III secretion system gene (*rhcN*) of strain DOA9. For this purpose, an internal fragment (263-bp) of *rhcN* was amplified by polymerase chain reaction (PCR) using the following primers: RhcN.D9p.int.f (5′-CATCTCGTCGACTCGCAGCAAAGGATGTCGATAC-3′) and RhcN.D9p.int.r (5′-GAGCAGTCTAGACCCGACTGACACTTCCTGCATG-3′). The PCR product was then digested by *Xba*l and *Sal*I and cloned onto the corresponding sites of the plasmid pVO155-Sm-npt2-gfp (Wongdee et al., 2016). This plasmid, which is non-replicative in *Bradyrhizobium* strains, is a derivative of the plasmid pVO155 (Oke and Long, 1999) and carries the promoterless *gusA* gene, a constitutively expressed gfp, and a Km- and a Sm/spectinomycin-resistance genes (Wongdee et al., 2016). The resulting plasmid was introduced into *E. coli* S17-1 by electroporation (15 kv/cm, 100 Ω, and 25 μF) and was transferred into the *Bradyrhizobium* sp. strain DOA9 by biparental mating, using the protocol described by Giraud et al. (2010). The transconjugants were spread on YM medium supplemented with an antibiotic mixture of Sm, Km, and Nal, and the successful insertion of the plasmid in *rhcN* was checked by PCR.

The GFP-labeled DOA9 strain was constructed by introducing the replicative plasmid pMG103-npt2-Sm-npt2-gfp harboring a constitutive *gfp* gene by electroporation (15 kv/cm, 100 Ω, and 25 μF). Plasmid-containing strains were selected on YM plates supplemented with Sm.

### Plant Nodulation and Symbiosis Analysis

DOA9 and its derivatives were grown for 5 days as previously described and used as inoculum. Sterilization and germination of the seeds of all plants tested (Supplementary Table S1) were performed as described by Teamtisong et al. (2014). Plants were sown in Leonard’s jar filled with sterilized vermiculite (Somasegaran and Hoben, 1994). All the plants were watered with BNM medium (Ehrhardt et al., 1992) and grown under the following controlled environmental conditions: 28 ± 2°C with a 16 h light/8 h dark cycle at light intensities of 300 μE/m^2^S and with 50% humidity. Five days after planting, each seedling was inoculated with 1 mL of a 5 day old inoculum (log-phase) after washing and adjusting the optical density to 600 nm to 1 (approximately 10^9^ cells). The experiment was conducted with five replicates with the following treatments: control (no inoculation), GPF-labeled DOA9, and Ω*rhcN*. To check nodulation and nitrogen fixation abilities, five plants were taken at 21 day post-inoculation (dpi) and the number of nodules and nitrogenase activity were assessed using the acetylene reduction assay (ARA). Briefly, all the nodules on each plant were collected and placed in headspace bottles with 10% acetylene, and incubated at 28°C for 1 h. Gas chromatography was used to measure the peak height of ethylene and acetylene with 1 mL gas samples from the bottles by using a PE-alumina packed column with injector at 150°C, an oven temperature of 200°C, and ionization detector (FID) at a temperature of 50°C (Somasegaran and Hoben, 1994).

### Rice Experiment

Rice (*Oryza sativa* L. ssp. *indica* cv. Pathum Thani) seeds were surface sterilized by soaking in 70% (v/v) ethanol for 30 s, then washed twice with 10% (v/v) hydrogen peroxide for 10 min, washed again in 3% (v/v) sodium hypochlorite for 1 h. Finally, the seeds were washed in sterilized water five times and germinated overnight on YM medium containing 0.8% (v/v) agar. The germinated seeds with 1 cm of root were soaked in DOA9 cell suspension (OD_600_ = 1) overnight. The rice seedlings were transferred to metal net in 80 mL tubes containing 15 mL Hoagland’s plant growth medium (Hoagland and Arnon, 1950) in presence of 0.1 mM NH_4_NO_3_. The experiment was conducted with three technical replicates with the following treatments: control (no inoculation), GFP-labeled DOA9, and Ω*rhcN*. Plants were grown under the following controlled environmental conditions: 28 ± 2°C with a 16 h light/8 h dark cycle at light intensities of 300 μE/m^2^S and with 50% humidity.

### Enumeration of Epiphytic and Endophytic Bacteria in Rice Roots

Two and 3 weeks after inoculation with the DOA9 tagged strains and Ω*rhcN* containing *gfp* and Sm^r^, the rice roots were washed in normal saline solution (0.85% of NaCl) to remove debris and any bacteria not firmly attached to the root surface. Next, the bacterial cells remaining on the surface of the roots were harvested by vortex with normal saline containing 0.3% (v/v) tween-80 for 30 s. The washing solution containing the epiphytic bacteria was then spread on YM plates containing Sm. For the enumeration of endophytic bacterial, root tissues were surface sterilized with 70% (v/v) ethanol for 5 min followed by 3% (v/v) sodium hypochlorite for 5 min and rinsed with sterilized water five times. The surface sterilized roots were macerated with a sterilized mortar and pestle and diluted in saline solution prior to being spread on medium as described above (Piromyou et al., 2015a). The epiphyte and endophyte population counted on agar plates are expressed as colony-forming unit per gram of root fresh weight.

### Preparation of Root Exudates and Bacterial Induction

The sterilized legume seeds were germinated and transferred into tubes containing BNM medium (50 mg seeds/mL). Plants were maintained in controlled environmental conditions as described above for 5 days. The root exudates were obtained from plant medium after filtration using a 0.2 μm filter syringe. Root exudates were stored at -20°C until use.

The mid-log phase culture of DOA9 was washed and the OD_600_ was adjusted to approximately 0.4 with YM supplemented with 1/3 (v/v) of the root exudates or purified flavonoids (20 μM of naringenin or genistein dissolved in DMSO). The sterilized BNM medium and DMSO were used as negative controls. The bacterial cells were cultured at 28°C for 24 h and then collected by centrifugation (4,000 × *g* for 10 min, 4°C) and immediately frozen in liquid nitrogen and stored at -80°C for further total RNA isolation.

### RNA Isolation and qRT-PCR

Total bacterial RNA was extracted from induced cells using the RNeasy^®^ Mini Kit (QIAGEN, United States) according to the manufacturer’s protocol. Total RNA was treated with RNase-free DNase I (NEB) for 30 min at 37°C. cDNA was synthesized from 500 ng total RNA using High Capacity cDNA Reverse Transcription Kits (Applied Biosystems^TM^) according to the manufacturers’ protocols. Thirty nanograms of cDNA was subjected to PCR amplification using specific primers of *rhcN* (rhcN.RT.D9.f: 5′-CATTGGCGATATGGTAGGCT-3′; rhcN.RT.D9.r: 5′-GGACAAGTGTGAACCGTCCT-3′) and *nopX* (nopX.RT.D9.f: 5′-CATCAACCCGAACAACACAG-3′; nopX.RT.D9.r: 5′-GGCTCGATAGACAAGGTCCACAA-3′) genes. PCR amplification was performed using QuantStudio 3 Real-Time PCR System Mix (Applied Biosystems^TM^) and the following program: an initial denaturation step at 95°C for 2 min; 35 cycles at 95°C for 30 s, 55°C for 30 s, and at 72°C for 1.5 min; and a final extension step at 72°C for 10 min. The relative gene expression was analyzed using the comparative Ct method (-ΔΔCT) normalized to the endogenous housekeeping gene (16S rRNA) using PBA338F (5′-ACTCCTACGGGAGGCAGCAG-3′) and PRUN518R (5′-ATTACCGCGGCTGCTGG-3′). Three biological replicates were pooled and analyzed. At least three replicate PCR amplifications were performed for each sample.

### Microscopy

An Olympus Fluoview FV1000 confocal laser scanning microscope was used to investigate nodule development, bacteroid differentiation, and rice colonization. For this purpose, 40–50 μm thick sections of fresh nodules and rice roots were prepared using a VT1000S vibratome (Leica Nanterre, France). Nodule sections were stained for 20 min with 0.01% calcofluor (to stain the plant cell wall) and with 30 μM propidium iodide (PI) in 50 mM Tris–HCl pH 7.0 buffer to identify dead cells (Haag et al., 2011). Calcofluor was excited at 405 nm and detected with a 460–500 nm emission filter. The GFP-labeled DOA9 and Ω*rhcN* strains were detected after excitation with the 488 nm laser line and emission signal collection at 490–522 nm, while the PI used to identify dead cells was excited with the 535 nm laser line and emission signals were collected at 617–636 nm. Confocal images were reconstructed with NIS elements software (Nikon), and images were colored and prepared for publication with Adobe Photoshop software. To detect β-glucuronidase (GUS) activity in the nodules elicited by the *rhcN* mutant the same protocol than Bonaldi et al. (2010) was used.

### Search for Genes Encoding Putative T3 Effectors on the DOA9 Genome

Two complementary approaches were used to identify putative effector genes: (i) a TBlastN search of the genome for known Nops identified in rhizobia (Kimbrel et al., 2013; Staehelin and Krishnan, 2015), the identified homologs were considered as possible candidates when they matched the following parameters (% of identity ≥40% over 80% of the length of the sequence) and (ii) a search for *tts*-boxes on the chromosome and plasmid of DOA9 genome (GenBank accession numbers DF820425 and DF820426, respectively) using a hidden Markov model initially trained with the sequences of 26 confirmed *tts-*boxes from *Sinorhizobium* (*Ensifer*) sp. strain NGR234, *B. diazoefficiens* USDA110, and *B. elkanii* USDA61 (Eddy, 1998; Marie et al., 2004; Zehner et al., 2008; Okazaki et al., 2009). To be considered as a candidate, the gene had to contain a *tts*-box with a score ≥9 located on the same strand in its upstream region (up to 1 kb).

### Statistical Analysis

The experiment data were subjected to analysis of variance (ANOVA). When confirming a statistically significant value in the *F*-test (*p* ≤ 0.05), a *post hoc* test (Duncan’s multiple-range test at *p* ≤ 0.05) was used as a multiple comparison procedure (Duncan, 1955) using SPSS1 software for WINDOWS^TM^, Version 14.0.

## Results

### Gene Organization and Comparison of the T3SS Genes of *Bradyrhizobium* sp. DOA9 and Other Rhizobial Strains

**FIGURE 1:**
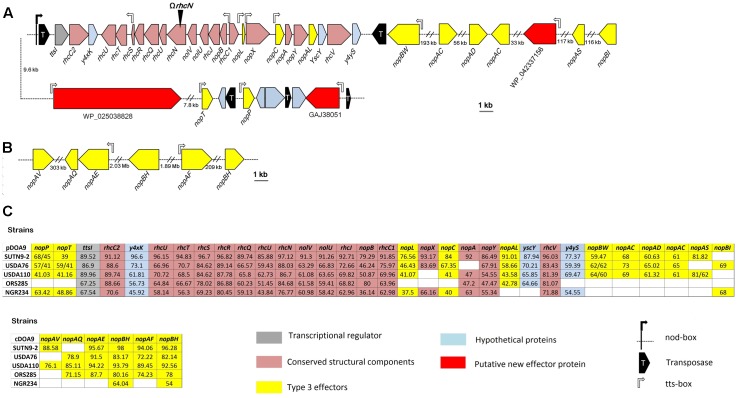
Genetic organization of the type 3 secretion gene (T3SS) cluster and putative effectors in *Bradyrhizobium* sp. DOA9 strain **(A)** on the plasmid pDOA9 and **(B)** on the chromosome. The orientations and sizes of the ORFs are indicated by arrows. Location and orientation of *tts*-box motifs are shown by rectangular open arrows, while the *nod*-box motif is shown by a black rectangular arrow. The site of insertional mutation for Ω*rhcN* is indicated by a black arrowhead. **(C)** % of similarity of the T3SS apparatus and effector proteins between DOA9 strain and other rhizobial strains, including *Bradyrhizobium* sp. SUTN9-2, *B. elkanii* USDA76, *B. diazoefficiens* USDA110, *Bradyrhizobium* sp. ORS285, and *Sinorhizobium fredii* NGR234.

Sequence genome analysis revealed the presence of a T3SS gene cluster organized in several operons on the pDOA9 plasmid (**Figures 1A,B**). The main putative operon preceded by a *tts*-box contained most of the genes coding for components of the secretion machinery in the order *nopB*, *rhcJ*, *nolU*, *rhcL*, *rhcN*, *rhcU*, *rhcQ*, *rhcR*, *rhcS*, *rhcT*, and *rhcU*. This genetic organization is similar to that found in other *Bradyrhizobium* or *Sinorhizobium* strains and a very high level of AA identity was observed with the corresponding homologous proteins. However, unlike these strains, a large intergenic region of 650 bp was observed between *rhcS* and *rhcT* also containing a *tts*-box (**Figure 1A**), raising the possibility that the *rhcT* and *rhcU* genes are transcribed independently.

As described in other rhizobia, we could assume that this main cluster is under the dual control of TtsI and NodD since a *ttsI* homolog was found at the vicinity of the main operon preceded by a *nod*-box (**Figure 1A**). However, it is to highlight the presence of a gene encoding a putative transposase between this *nod*-box and *ttsI*. To confirm that the presence of this putative transposase does not interfere with the formation of the T3SS secretion apparatus, we analyzed the expression of two structural T3SS genes (*rhcN* and *nopX*) in the presence of two flavonoids (genistein or naringenin) previously reported to induce T3SS genes in rhizobial strains (Krause et al., 2002; Okazaki et al., 2010). As shown in Supplementary Figure S1, both flavonoids strongly induced *nopX* and *rhcN* expression, showing that despite this transposase, the T3SS genes in DOA9 strain are classically regulated by flavonoids, most probably via the control of NodD and TtsI.

To identify putative effectors that are possibly translocated by this T3SS machinery, we combined two *in silico* searches: (i) a TBlastN search of the genome for Nops previously identified in other rhizobia and (ii) a search of the *tts*-box motif using a hidden Markov model. After eliminating the Nops proteins predicted to correspond to components of the secretory apparatus (NopA, NopX, and NopB) (Staehelin and Krishnan, 2015), this analysis retrieved 20 putative translocated effectors. Of these, six were located on the chromosome (**Figure 1C**). However, all six candidates remain doubtful because homologous proteins for all of them can be identified in photosynthetic *Bradyrhizobium* strains that lack a T3SS gene cluster (Supplementary Table S2). Interestingly, among the other putative effectors found on the plasmid, three correspond to new effectors not previously identified in other rhizobia. They were preceded by a *tts*-box and all contained the small ubiquitin-like modifier (SUMO) protease domain of the C48 peptidase [ubiquitin-like protease 1 (Ulp1)] family. Several effectors containing this functional domain have already been characterized in pathogenic and symbiotic bacteria (Hotson et al., 2003; Hubber et al., 2004; Rodrigues et al., 2007; Tsurumaru et al., 2015), reinforcing the hypothesis that these three effector candidates are *bona fide* new effectors.

### Symbiotic Role of the T3SS

To analyze the role of the T3SS in DOA9 strain during symbiosis, we constructed a T3SS mutant by inserting the non-replicative plasmid pVO155-Sm-npt2-gfp into the 5′-region of *rhcN*. The *rhcN* gene was selected as target because it encodes an ATPase that is indispensable for the functioning of the T3SS injectisome. Furthermore, the pVO155 plasmid used for inactivation of the gene contains a constitutive expressed GFP that enables monitoring of the bacteria inside the nodules. The symbiotic performance of the WT and Ωr*hcN* mutant was compared on several legume species belonging to Genistoid (*Crotalaria juncea*), Dalbergioid (*Aeschynomene americana*, *A. afraspera*, *Arachis hypogaea*, and *Stylosanthes hamata*), and Millettioid tribes (*Macroptillium atropurpureum*, *Vigna radiata*, *Indigofera tinctoria*, and *Desmodium tortuosum*) by analyzing several symbiotic parameters (number of nodules per plant, nitrogen fixation estimated using the ARA, plant dry weight, and cytological aspect of the nodules).

**FIGURE 2:**
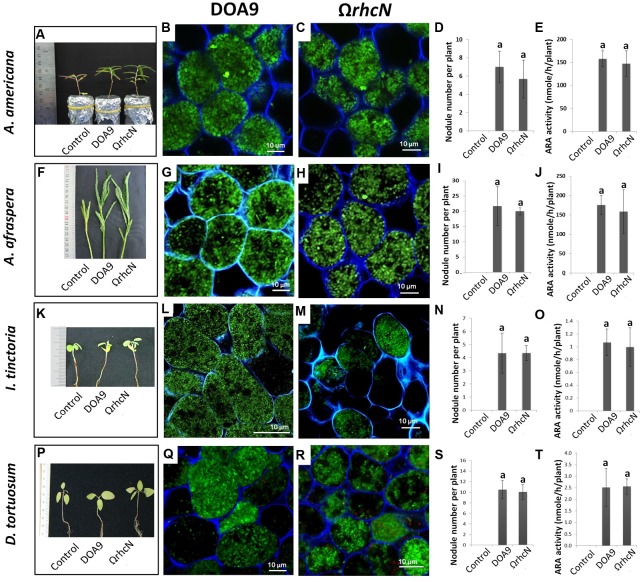
Symbiotic analysis of legume species displaying no phenotype with the T3SS mutant of the DOA9 strain. Plant growth, cytological aspect of the nodules, number of nodules per plant, and the ARA activity of *A. americana* **(A–E)**, *A. afraspera* **(F–J)**, *I. tinctoria* **(K–O)**, and *D. tortuosum* **(P–T)** after inoculation with the WT strain and the Ω*rhcN* mutant. Values represent mean ± SD (*n* = 5). Within treatment, means labeled with different letters are statistically different at *p* < 0.05.

Depending on the plant species, the Ω*rhcN* mutant displayed contrasted responses compared to the WT strain. The responses were classified in three categories. In category 1, non-responsive phenotype (called T3SS-no effect group), the plants inoculated with the mutant displayed the same number of nodules and the same nitrogen fixing capacity as the plants inoculated with the WT strain. Furthermore, no cytological differences were observed between the mutant and WT nodules. These plants were *A. americana*, *A. afraspera*, *I. tinctoria*, and *D. tortuosum* (**Figure 2**).

**FIGURE 3:**
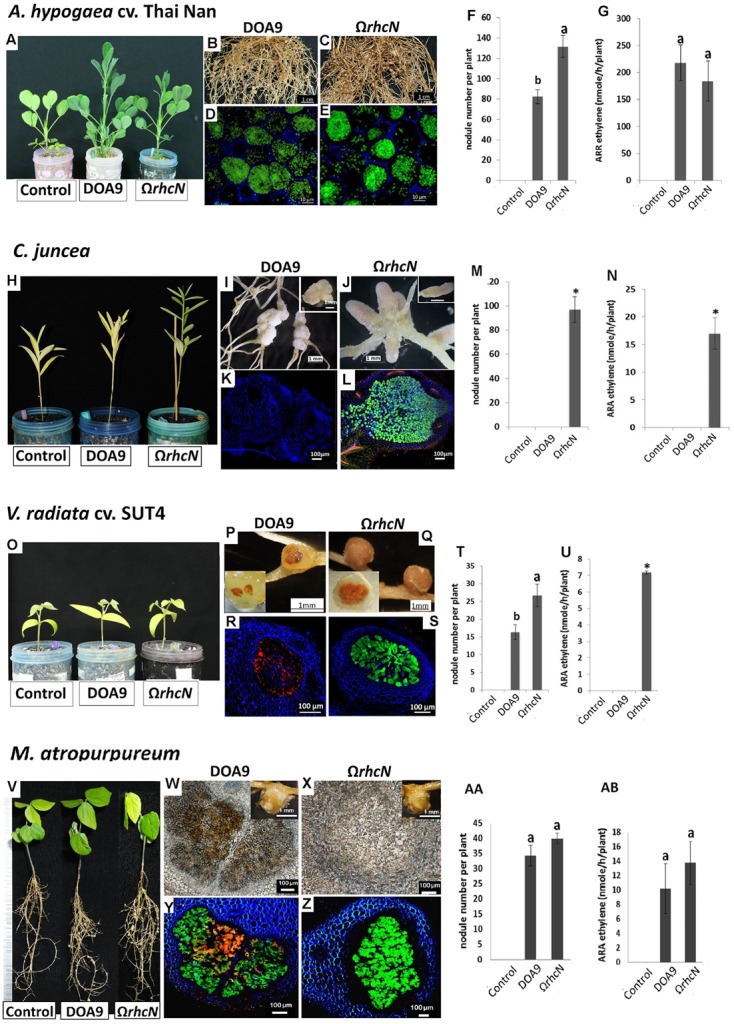
Symbiotic analysis of legume species displaying a positive phenotype with the T3SS mutant of DOA9 strain. Plant growth, cytological aspect of the nodules, number of nodules per plant, and the ARA activity of *A. hypogaea* cv. Thai Nan **(A–G)**, *C. juncea* **(H–N)**, *V. radiata* cv. SUT4 **(O–U)**, and *M. atropurpureum* (siratro) **(V–AB)** after inoculation with the WT strain and the Ω*rhcN* mutant. In **(D**,**E**,**L**,**R**,**S**,**Y**,**Z)**, dead cells are stained red with propidium iodide (PI). The green cells are viable cells labeled with GFP. Values represent mean ± SD (*n* = 5). Within treatment, means labeled with different letters are statistically different at *p* < 0.05.

In category 2, the *rhcN* mutation had a positive effect on one of the symbiotic parameters analyzed (**Figure 3**). This group comprised *A. hypogaea*, *V. radiata*, *C. juncea*, and *M. atropurpureum*. Inoculation with the Ω*rhcN* mutant led to an almost 65% increase in the number of nodules on *A. hypogea* compared with WT (**Figure 3F**). This result suggests that in DOA9, T3SS compromises either the infection or nodule organogenesis process in this species. **Figure 3** shows that the higher number of nodules is not correlated with an increase in N_2_ fixation. We assume that the plant compensates for the small number of nodules elicited by the WT strain by stimulating their expansion, as can be seen in **Figures 3B,C**. The same mechanism has been reported in *Medicago* and is related to plant N demand (Laguerre et al., 2012). The deleterious effect of the T3SS on the establishment of the symbiosis was more marked in the cases of *C. juncea* and *V. radiata*. Indeed, whereas the WT strain only elicited bumps or small completely necrotic nodules in *C. juncea* and *V. radiata*, respectively, the Ω*rhcN* mutant formed perfectly developed nodules on these two plants (**Figures 3H–U**). This suggests that some effectors translocated by the T3SS could activate plant defense reactions in these two species, thereby preventing infection and the development of nodules. Similar stimulation of plant immunity probably also occurred in *M. atropurpureum* but to a lesser extent, since the nodules induced by the WT strain were well formed, but displayed brown necrotic areas. The bacteroids in these areas were dead as revealed by the red PI staining, whereas the Ω*rhcN* nodules were perfectly normal and the host cells were filled with viable bacteroids, as revealed by the green of GFP tagging cells (**Figures 3V–AB**). Nevertheless, while the T3SS mutation made it possible to restore nodule formation capacity in *V. radiata* and *C. juncea*, only very weak nitrogenase activity was detected, and no real benefit for plant growth was observed (**Figures 3N,U** and Supplementary Table S3). This shows that other restrictions exist between DOA9 and these two plant species resulting in inefficient symbiosis.

**FIGURE 4:**
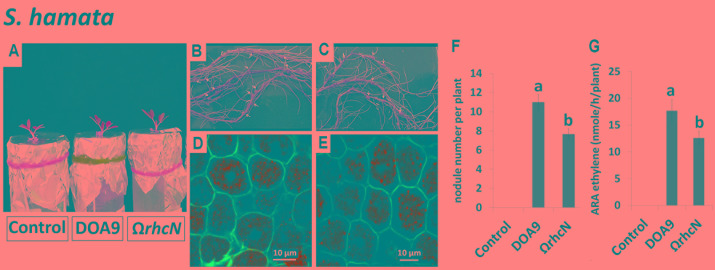
Symbiotic analysis of legume species displaying a negative phenotype with the T3SS mutant of DOA9 strain. Plant growth, cytological aspect of the nodules, the number of nodules per plant, and ARA activity of *S. hamata* **(A–G)** after inoculation with the WT strain and the Ω*rhcN* mutant. Values represent mean ± SD (*n* = 5). Within treatment, means labeled with different letters are statistically different at *p* < 0.05.

Only one species, *S. hamata*, was attributed to category 3, corresponding to a negative effect of the *rhcN* mutation. The number of nodules and nitrogenase activity of nodules induced by the Ω*rhcN* strain were reduced by approximately 30% compared with the wild-type strain (**Figures 4A–G**). These data suggest that the T3SS of strain DOA9 also plays a positive role in the establishment of the symbiosis, most probably by translocating effectors that weaken the plant immune system.

We also examined the effect of root exudates on the expression of *rhcN* and *nopX* genes to determine whether the absence of phenotype observed for the *rhcN* mutant in some legume species could be linked with the absence of formation of the T3SS apparatus. As expected, a significant level of expression of the two T3SS genes was detected in the five species displaying a positive or negative phenotype with the Ω*rhcN* strain (Supplementary Figure S1). In contrast, of the four species that displayed no phenotype with the *rhcN* mutant, the root exudates of two of them (*I. tinctoria* and *D. tortuosum*) displayed a similar level of expression to the control without inducers raising the possibility that the T3SS machinery is not formed during the early steps of the interaction between the DOA9 strain and these two legumes species. We also took advantage of the promoterless *gusA* reporter gene present in the insertional pVO155 plasmid used to construct the *rhcN* mutant to determine whether the T3SS is expressed in mature nodules as already reported in *B. diazoefficiens* USDA110 nodulating soybean (Zehner et al., 2008). As shown in Supplementary Figure S2, for the three tested species (*A. americana*, *M. atropurpureum*, and *I. tinctoria*), a GUS activity was detected in the infected nodule cells, in contrast to the WT nodules, in which no activity was observed (Supplementary Figure S2). Altogether, these data suggest that T3SS genes of DOA9 strain are active during the early and/or late stages of symbiosis on the different species tested.

### Role of the T3SS in Rice Colonization and Infection

**FIGURE 5:**
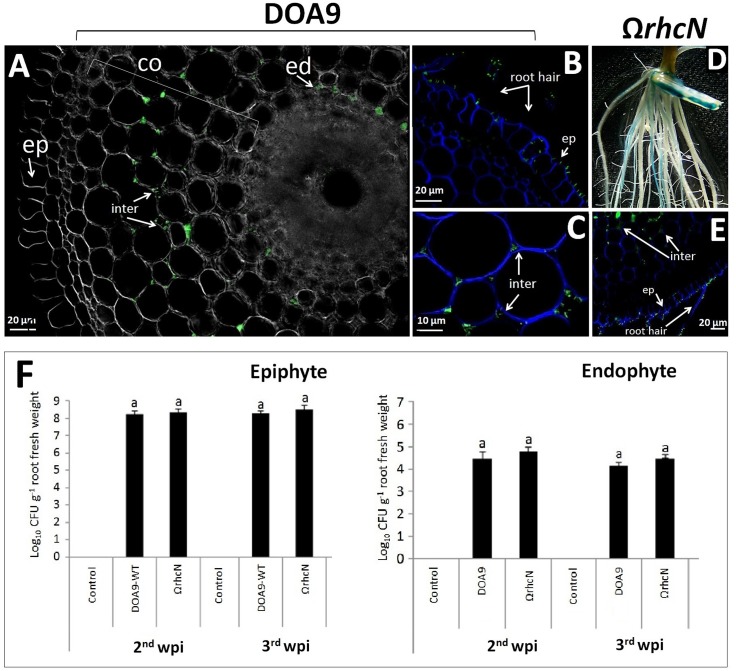
Ability of DOA9 strain and its derivative T3SS mutant to colonize rice roots. Endophytic colonization of the rice roots by *Bradyrhizobium* sp. DOA9-WT **(A–C)** and T3SS mutant (Ω*rhcN*) **(D,E)** 24 h after inoculation. **(D)** Expression of the *gusA* reporter gene inserted in *rhcN* revealed on rice roots [2 week post-inoculation (wpi)] stained with X-Gluc after 3 h of incubation. The intercellular spaces (inter) of the cortex (co) and endodermis (ed) are occupied by endophytic cells. **(F)** Bacteria colonizing the root surface (epiphyte) and root tissue (endophyte) at 2 and 3 wpi with *Bradyrhizobium* sp. DOA9 and T3SS mutant (Ω*rhcN*) strains. Values represent mean ± SD (*n* = 3). Within treatment, means labeled with different letters are statistically different at *p* < 0.05.

We also investigated the effect of the T3SS mutation on the ability of DOA9 to colonize the surface of rice roots and to infect deep root tissue. These observations were made on a Thai rice (cultivar Pathum Thani 1) at the second and third week post-inoculation (wpi) (**Figure 5F**). The total population density of WT and Ω*rhcN* strains on the surface of the rice roots was almost the same (around 8 log_10_ CFU/g of root fresh weight), and this level of population remained constant at 2 and 3 wpi. When the roots were surface sterilized to conserve only the endophytic bacteria cells, the estimated population of both WT and mutant strains was also very close (around 4 log_10_ CFU/g root fresh weight). We also took advantage of the GFP tag added on the WT and Ω*rhcN* strains to monitor invasion of the roots. As can be seen in **Figures 5A–C,E**, both WT and Ω*rhcN* cells were attached to the root hairs and to the surface of the root epidermis. Analysis of root sections revealed endophytic bacterial cells in the intercellular space of the root cortex and endodermis. Taken together, these data suggest that the T3SS mutation did not affect the ability of the DOA9 strain to colonize and infect the rice root tissue intercellularly. This absence of effect of the T3SS mutation was not related to the absence of T3SS genes expression considering that a GUS activity could be detected at the surface of the rice roots inoculated with the *rhcN* mutant (**Figure 5D**).

## Discussion

The *Bradyrhizobium* sp. DOA9 strain isolated from a paddy field has been shown to have the ability to induce the development of symbiotic nodules in various legume species (Teamtisong et al., 2014). Unlike other bradyrhizobia, this strain contains a symbiotic megaplasmid (pDOA9) that harbors the *nod* and *nif* genes as well as a T3SS gene cluster (Okazaki et al., 2015). This T3SS cluster contains all the genes necessary for the formation of the secretory apparatus and the transcriptional activator (TtsI), which is preceded by a *nod*-box promoter motif. This genetic organization is similar to that described in other bradyrhizobial strains. Why DOA9 harbors a symbiotic plasmid while all the other *Bradyrhizobium* strains sequenced contain a symbiotic island remains an open question. In both cases, the lower GC content and the codon usage of the genes present in the symbiotic region suggest an acquisition by lateral transfer. This indicates that, in addition to the possibility of a difference in the origin of the symbiotic region, the mechanisms of its transfer and its maintenance inside the bacterial genome could also differ from one *Bradyrhizobium* strain to another.

Despite high conservation of T3SS genes encoding the injectisome, the T3SS effector contents of DOA9 strain differ from those in other bradyrhizobia, notably in their number, which is comparatively lower. Indeed, in the DOA9 strain, only 14 candidate effector proteins have been predicted versus between 30 and 35 putative effectors in other bradyrhizobia (Zehner et al., 2008; Kimbrel et al., 2013). In particular, the effectors NopM and NopE, classically found in *Bradyrhizobium* strains, often as multicopies, are clearly absent in the DOA9 genome. Conversely, three putative effectors that were not previously identified in rhizobia are predicted in the DOA9 strain. These three new putative effectors proteins (WP_042337156, WP_025038828, and GAJ3805) most probably correspond to SUMO peptidases that contain the catalytic domain of members of the family of C48 cysteine proteases involved in the de-ubiquitinization of eukaryotic proteins (Hotson et al., 2003). Interestingly, a SUMO peptidase protein named NopD in *S. fredii* HH103 was identified to be secreted through the T3SS in the culture supernatant indicating that this family protein could constitute *bona fide* effectors translocated into the host cell (Rodrigues et al., 2007). Furthermore, more recently, other SUMO protease putative effectors were reported to be responsible for the incompatibility of some *Bradyrhizobium* strains (*B. japonicum* Is-1 and *B. elkanii* USDA61), which prevents them from interacting symbiotically with Rj4 genotype soybeans (Faruque et al., 2015; Tsurumaru et al., 2015). Taken together, this reinforces the hypothesis that the three new Nop candidats identified in DOA9 strain are effectors that could also affect the ability to interact with some plants.

It has previously been shown that T3SS in bradyrhizobia can have a positive or negative effect on nodule formation by stimulating or repressing the plant immune system depending on the host plant. For example, the deletion of T3SS in *B. diazoefficiens* delayed nodulation on soybean and reduced the number of nodules on *M. atropurpureum* (Krause et al., 2002), while the absence of T3SS in *B. elkanii* USDA61 induced the formation of nodules on *V. radiata* L. cv. KPS1 and on the soybean cultivar Hill, which contains the Rj4 allele (Okazaki et al., 2009). In the present study, we observed that the T3SS mutation affected the ability of DOA9 strain to interact with five out of the nine species tested. In particular, the T3SS mutation dramatically improved nodule formation and development in *C. juncea* and *V. radiata* and had a more moderate but also positive impact on nodulation in *A. hypogaea* and *M. atropurpureum*. This suggests that among the cocktail of effectors that are translocated into the host cell, some are recognized as virulence factors by R-proteins and block infection and nodule organogenesis to a greater or lesser extent. As previously mentioned above, some SUMO protease effectors and also NopT have been observed to act as negative effectors in some hosts (Dai et al., 2008), which raises the possibility that the effector(s) responsible for this incompatibility could be one or some of the new effectors identified in the DOA9 strain or even NopT, which is also present in DOA9.

Conversely, the T3SS mutation had a negative impact on the number of nodules elicited on *S. hamata*, suggesting, in this case, that some effectors act positively on the symbiosis most probably, either by helping the bacteria to overcome plant defense reactions that limit the interaction or by directly inducing the nodulation signaling pathway as recently described for some non-photosynthetic bradyrhizobia shown to nodulate some legume species in the absence of NFs synthesis but thanks to the T3SS (Okazaki et al., 2013, 2016).

Identifying the effectors responsible for these contrasted symbiotic effects depending on the host plant, and understanding their role are real challenges. To date, a specific role in the symbiosis has been described for only a few effectors (Staehelin and Krishnan, 2015). One limitation is that the different effectors translocated into the host cell mostly act synergistically by interfering in different plant defense pathways. The DOA9 strain may be an ideal model to study the specific role of effectors individually or in combination. Indeed, all the symbiotic determinants are found on the pDOA9 plasmid and it has been possible to create a plasmid-free DOA9 strain (Tittabutr, personal communication). We can imagine designing plasmids encompassing a minimum set of canonical *nod*, *nif*, and T3SS injectisome genes supplemented by different combinations of effectors and to study the symbiotic properties of a DOA9 strain lacking pDOA9 in which these synthetic plasmids are re-introduced. This kind of approach may seem too ambitious and time consuming, but with the development of synthetic biology and a reduction in the cost of synthetizing DNA, it could be feasible in the near future.

Among the four species in which the T3SS mutation had no effect on the symbiosis, we observed that the root exudate of two of them (*I. tinctoria* and *D. tortuosum*) did not induce the expression of *nopX* and *rhcN* genes, suggesting that the T3SS apparatus is not formed during the early stages of the symbiosis between the DOA9 strain and these two species. These results were surprising considering that these two species were found to be nodulated by the DOA9 strain, indicating that the *nod* genes are expressed. It would be interesting to explore more deeply the regulation of the T3SS and *nod* genes in presence of flavonoids and root exudates to determine if decoupling between their expression is possible. In the same vein, we observed that some putative T3SS effector genes are not preceded by a *tts*-box, which raises the question of whether these genes are expressed when the T3SS is formed or if their regulation involves another activator than TtsI. The observation that T3SS genes were found expressed in mature nodules while they were not found activated by root exudates is also puzzling. This could be related to the fact that we used different technics to analyze genes expression or that the technic of extraction of root exudate was not adapted for *I. tinctoria* considering that this very small plant do not grow well in liquid medium. We cannot also exclude the possibility that the regulation of the T3SS genes could be more complex involving other input signals than flavonoids and additional regulators than NodD and TtsI leading to different gene expression controls during the early and late stages of symbiosis.

Finally, given that a previous study focusing on the SUTN9-2 strain showed that the T3SS is an important determinant in rice infection (Piromyou et al., 2015b), we also investigated if the T3SS plays a similar role during the interaction between the DOA9 strain and rice. However, despite the use of the same cultivar (Pathum Thani 1), our observations indicate that the T3SS mutation does not affect the ability of DOA9 strain to colonize the root surface and to infect the root tissue intercellularly. This result was surprising given that the two strains were isolated from similar ecological niches (Thai paddy fields using *A. americana* as trap legumes) and considering that we observed that the T3SS genes are active during the interaction of DOA9 strain with rice. These two strains differ in the origin of the symbiotic region, which harbors a different cortege of *nod* and *nop* genes. We can suppose that during evolution, each strain adapted this symbiotic toolbox differently as a function of the variety of plant partners it encountered during its life history. This might explain why, via a different cocktail of effectors, the same T3SS machinery can lead to different responses from one strain to another and from one host to another.

## Author Contributions

PS, PT, NB, EG, and NT conceived the experiment(s). PS, RN, KT, JP, AT, PT, and PP conducted the experiment(s). PS, PT, EG, and NT analyzed the result(s) and wrote the paper. All authors reviewed the manuscript.

## Conflict of Interest Statement

The authors declare that the research was conducted in the absence of any commercial or financial relationships that could be construed as a potential conflict of interest.
