# Supplementary material for: Type 3 Secretion System (T3SS) of Bradyrhizobium sp. DOA9 and Its Roles in Legume Symbiosis and Rice Endophytic Association
Source: Front Microbiol. 2017 Sep 20;8:1810. doi: 10.3389/fmicb.2017.01810 (PMC5611442; doi:10.3389/fmicb.2017.01810)
Supplement: Supplementary file 1 [file Table_1.docx]

Supplementary Material

Type 3 Secretion System (T3SS) of *Bradyrhizobium* sp. DOA9 and its Roles in Legume Symbiosis and Rice Endophytic Association

**Pongpan Songwattana^1^, Rujirek Noisangiam^2^, Kamonluck Teamtisong^3^, Janpen Prakamhang^4^, Albin Teulet^5^, Panlada Tittabutr^1^, Pongdet Piromyou^1^, Nantakorn Boonkerd^1^, Eric Giraud^5*^, Neung Teaumroong^1*^**

* **Corresponding author**: [neung@sut.ac.th](mailto:neung@sut.ac.th) *and* eric.giraud@ird.fr
Note: EG and NT contribute equally to this work

| **Tribes** | **Plants tested** | **Source or references** |
| --- | --- | --- |
| Gienistioid | *Crotalaria juncea* | Nakhon Rathasima, Thailand |
| Derbergioid | *Aeschynomene americana*  *Aeschynomene afraspera*  *Arachis hypogaea* cv. Thai Nan  *Stylosanthes hamata* | Nakhon Rathasima, Thailand  LSTM, IRD, France  Nakhon Rathasima, Thailand  Nakhon Rathasima, Thailand |
| Millettioid | *Macroptilium atropurpureum*  *Indigofera tinctoria*  *Vigna radiata* cv. SUT4  *Desmodium tortuosum* | Nakhon Rathasima, Thailand  Nakhon Rathasima, Thailand  Nakhon Rathasima, Thailand  Nakhon Rathasima, Thailand |
| Non-leguminous plant | *Oryza sativa* L. cv. Pathum Thani1 | Department of Agriculture, Thailand |

# Supplementary Tables

**Table S1** Plants tested in this study

36

**Table S2** List of putative Nop effectors and *tts*-boxes identified in *Bradyrhizobium* DOA9 strain

| **Gene ID**^a^ | **Replicon**^b^ | **Protein homology**^c^ | **Upstream *tts-box* sequence**^d^ | **Characteristics of proteins**^e^ |
| --- | --- | --- | --- | --- |
| *WP_025038413* | P | NopBI (K) | ni. | put. kinase Y4dM (HipA-like protein) |
| *WP_025038507* | P | NopAS (K) | ni. | put. panB |
| WP_042337156 | P | put. new Nop | TB1. **GTCAG**CATCTC**G**TA**AG**TTTGACCGAGT**A** | C-terminal C48 (SUMO) peptidase domain |
| GAJ37770 | P | NopAC (S) | ni. | put. endo-polygalacturonase |
| GAJ37771 | P | NopAD (K) | ni. | put. pectinesterase |
| WP_025038647 | P | NopAC (S) | ni. | put. endo-polygalacturonase |
| WP_025038801 | P | NopBW (S) | TB2. **GTCAG**CCAACC**G**TC**AG**CCAGGCGGCCT**A** | unknown |
| WP_042337078 | P | NopC (S) | TB3. **GTCAG**C**T**TGTC**G**AA**AG**CTAATACTTGC**A** | secreted effector |
| WP_082848019 | P | NopA (S) | TB3. | T3SS pili component |
| WP_025038808 | P | NopAL (K) | TB3. | unknown |
| WP_025038811 | P | NopX (S) | TB4. **GTCAG**C**T**TTTG**G**AA**AG**CTAATCGAACCT | NolX : T3SS translocon |
| GAL91145 | P | NopL (S) | TB5. **GTCAG**C**T**TTTC**G**AA**AG**CGACTGCTGCC**A** | secreted effector |
| GAJ38008 | P | NopB (S) | TB6. **GTCAG**T**T**TTTG**G**TC**AG**CTTGTTTCTCT**A** | T3SS pili associated Nop |
| WP_025038820 | P | RhcT | TB7. **GTCAG**C**T**TATC**G**AA**AG**CTCGCCTCACC**A** | T3SS minor apparatus component |
| WP_025038828 | P | put. new Nop | TB8. **GTCAG**C**T**TCAA**G**TC**AG**GTTAGAACAGT**A** | C-terminal C48 (SUMO) peptidase domain |
| WP_025038834 | P | NopT (S) | TB9. **G**A**CAG**C**T**TGCC**G**TC**AG**CTTGGCCAAGT**A** | cysteine protease - secreted effector |
| GAJ38044 | P | NopP (S) | TB10. **GTCAG**GCTCCG**G**AC**AG**CTAAGCTGTCT**A** | secreted effector |
| GAJ38051 | P | put. new Nop | TB11. **GTCAG**CCTGTC**G**TC**AG**GCTGCAACGGT**A** | C-terminal C48 (SUMO) peptidase domain |
| *WP_025034134* | C | NopAF (K) | TB12. **GTCAG**T**T**TGTC**G**TG**AG**TTGTCCCGGGT**A** | put. transpeptidase |
| *WP_025033944* | C | NopBH (K) | ni. | put. aminopeptidase |
| *WP_025035848* | C | NopBH (K) | ni. | put. aminopeptidase |
| *WP_025037732* | C | NopAE (K) | TB13. **GTCAG**G**T**TTCC**G**TC**AG**GTGACCGCCGAC | put. quinoprotein ethanol dehydrogenase |
| *WP_025037734* | C | NopAQ (K) | TB13. | put. ABC transporter periplasmic binding protein |
| *WP_025037995* | C | NopAV (K) | ni. | put. carboxylesterase |

^a^ in italics, these candidate genes are considered as doubtful because homologs are also found in *Bradyrhizobium* strains lacking a T3SS. ^b^ P, genes located on the plasmid; C, genes located on the chromosome. ^c^ (S) and (K) are used to indicate that Nop names were attributed according to the nomenclature used in the publication of Staehelin and Krishnan, 2015 (S) and Kimbrel *et al.*, 2013 (K); put. indicated putative; ^d^ in bold are indicated the nucleotides that are conserved with the consensus *tts-box* motif described in Zehner *et al.*, 2008. ni., *tts-boxes* not identified in the upstream region of the gene; ^e^ put. indicated putative.

**Table S3** Total plant dry weight (mg) of all plants tested after inoculated with *Bradyrhizobium* sp. DOA9 and mutant Ω*rhcN* strain

| **Tested plants / strains** | | **Total plant dry weight (mg)** | | |  |
| --- | --- | --- | --- | --- | --- |
|  |  | **Control^1^** | **DOA9** | ***ΩrhcN*** | |
| Genistoid | *C. juncea* | 169±20 ab^2^ | 152±4.9 a | 196±38.3 b | |
| Dalbergioid | *A. americana* | 13.1±2.2 a | 67.4±4.9 b | 53.6±9.9 b | |
|  | *A. afraspera* | 54.4±7.9 a | 78.2±6.9 b | 79.2±2.8 b | |
|  | *A. hypogaea*  cv. Thai Nan | 1200±219 a | 2112±330 b | 1779±248 b | |
|  | *S. hamata* | 5.0±0.6 a | 7.8±1 c | 6.4±1.1 ab | |
| Millettioid | *M. atropurpureum* | 55.9±3.2 a | 59.2±3.1 a | 60.2±5.6 a | |
|  | *I. tinctoria* | 5.1±0.6 a | 5.1±0.7 a | 5.0±0.8 a | |
|  | *V. radiata* cv. SUT4 | 45.6±9.0 a | 47.2±9.3 a | 45.6±13.4 a | |
|  | *D. tortuosum* | 6.5±1.2 a | 6.3±1.1 a | 7.3±1.1 a | |

Values are shown as Mean±SD of at least triplicates, ^1^ : uninoculation control, ^2^ a-d means in a column followed by the same letter are not significantly different by Dancan’s multiple range test at the 5% (p≤0.5), ^3^ means not observed in this study.
