# Supplementary figures and images for: Type 3 Secretion System (T3SS) of Bradyrhizobium sp. DOA9 and Its Roles in Legume Symbiosis and Rice Endophytic Association
Source: Front Microbiol. 2017 Sep 20;8:1810. doi: 10.3389/fmicb.2017.01810 (PMC5611442; doi:10.3389/fmicb.2017.01810)

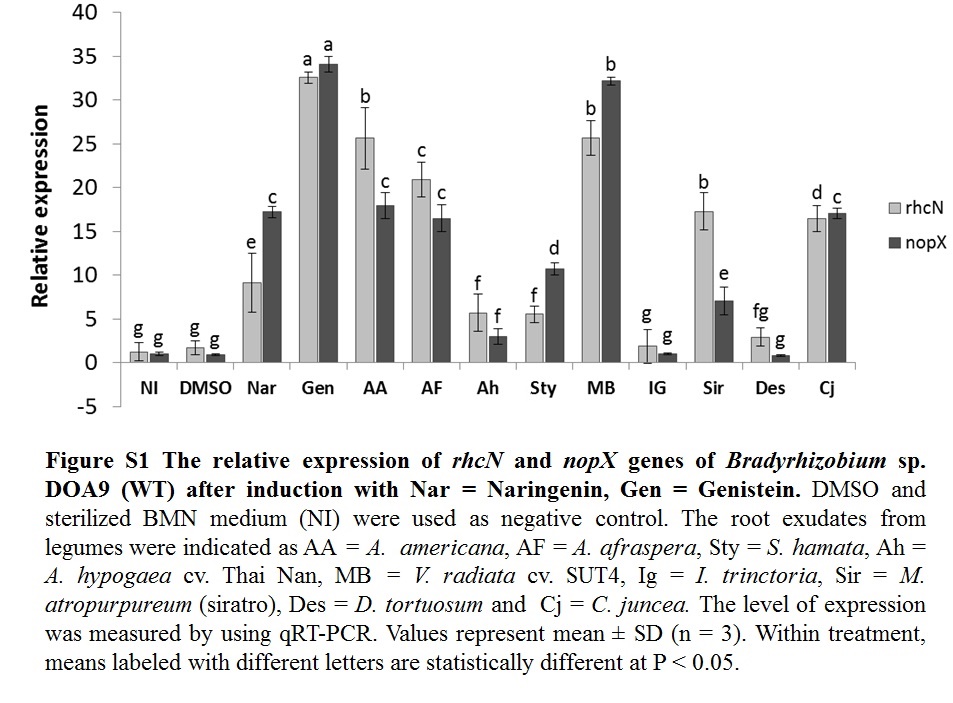

Supplement: Supplementary file 2 [file Image_1.jpg]

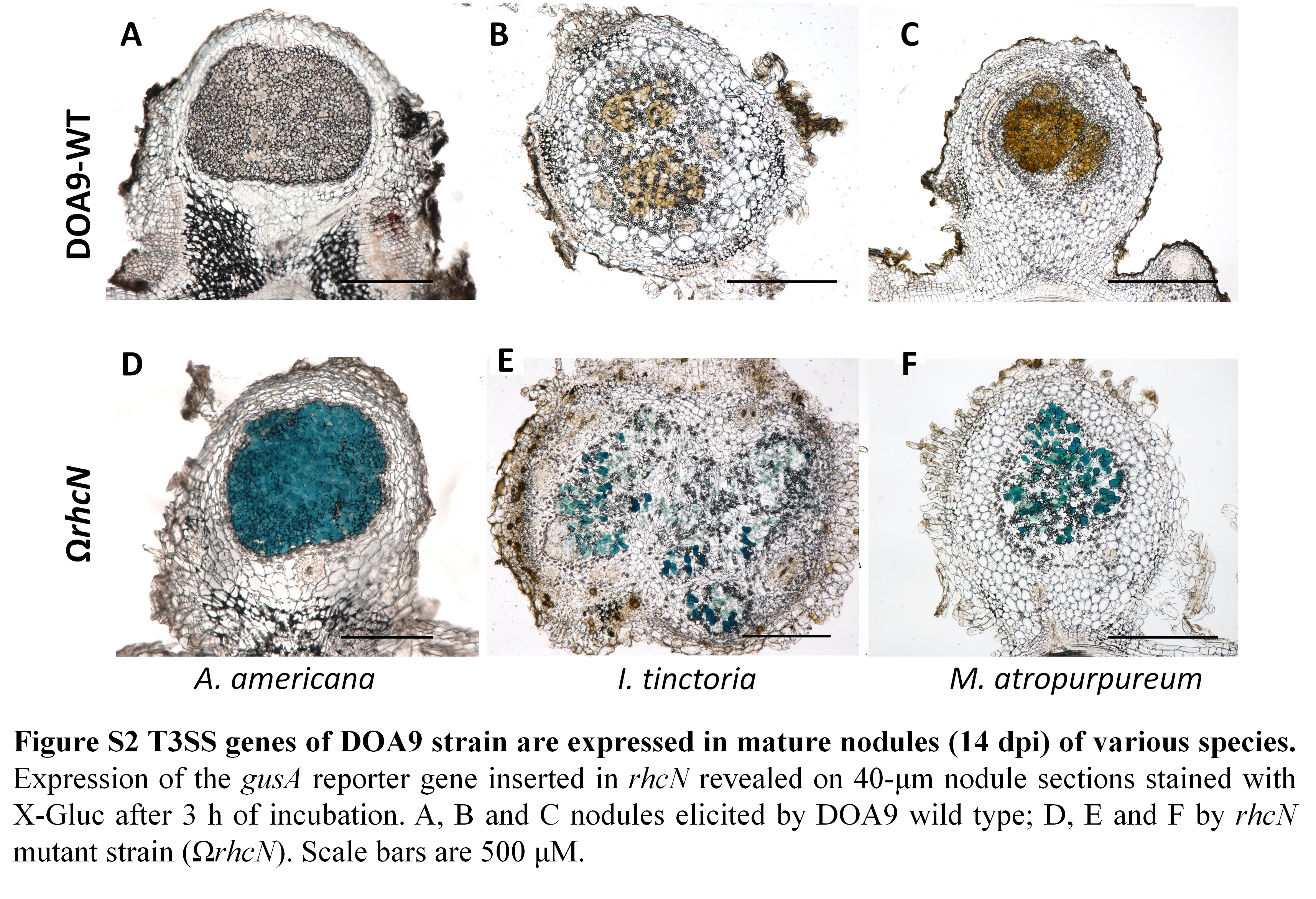

Supplement: Supplementary file 3 [file Image_2.JPEG]
